# Analysis of *MMP-2*-735C/T (rs2285053) and *MMP-9*-1562C/T (rs3918242) Polymorphisms in the Risk Assessment of Developing Lung Cancer

**DOI:** 10.3390/ijms241310576

**Published:** 2023-06-24

**Authors:** Katarzyna Wadowska, Piotr Błasiak, Adam Rzechonek, Mariola Śliwińska-Mossoń

**Affiliations:** 1 Department of Medical Laboratory Diagnostics, Division of Clinical Chemistry and Laboratory Haematology, Wroclaw Medical University, Borowska 211A, 50-556 Wroclaw, Poland; katarzyna.wadowska@student.umw.edu.pl; 2 Department and Clinic of Thoracic Surgery, Wroclaw Medical University, Grabiszyńska 105, 53-439 Wroclaw, Poland; blasiakpiotr@gmail.com; 3 Department of Thoracic Surgery, Lower Silesian Centre of Oncology, Lung Diseases and Haematology, Grabiszyńska 105, 53-439 Wroclaw, Poland; adam.rzechonek@gmail.com

**Keywords:** lung cancer, polymorphism, matrix metalloproteinases, *MMP-2*-735C/T, rs2285053, *MMP-9*-1562C/T, rs3918242, ethnicity

## Abstract

Matrix metalloproteinase (MMP)-2 and -9 are gelatinases which are capable of degrading type IV collagen and have been linked to cancer invasion and metastatic development. MMP-2 and MMP-9 gene polymorphisms may affect their biological function, and thus their role in cancer development and progression. We analyzed the association of the polymorphism frequencies of *MMP-2*-735C/T and *MMP-9*-1562C/T with MMP-2 and MMP-9 serum concentrations, as well as their potential effects in lung cancer patients. We conducted a retrospective, case-control study consisting of 112 lung cancer patients and 100 healthy individuals from a Caucasian population in Poland. Polymerase chain reaction with restriction fragment length polymorphism (PCR/RFLP) and electrophoresis was used to genotype genomic DNA from whole blood samples. MMP-2 and MMP-9 serum concentrations were then determined using ELISA. For statistical analysis, Statistica version 13 from TIBCO Software Inc. was utilized with a significance level <0.05. Logistic regression analysis revealed that *MMP-2*-735CC (OR = 5.39; 95% CI = 0.62–47.17; *p* = 0.238504) and -735CT genotype (OR = 7.22; 95% CI = 0.78–67.14; *p* = 0.072836), as well as *MMP-9*-1562CC (OR = 1.45; 95% CI = 0.31–6.70; *p* = 0.757914) and -1562CT genotype (OR = 1.60; 95% CI = 0.33–7.83; *p* = 0.548801) were associated with a higher risk of lung cancer. There were statistically significant differences observed in the MMP-2 concentration between individuals with the -735CC genotype and the -735CT genotype (non-smoking control: 204.04 ng/mL vs. 237.00 ng/mL, respectively, *p* = 0.041479; adenocarcinoma patients: 157.69 ng/mL vs. 126.37 ng/mL, respectively, *p* = 0.013222), as well as differences in the MMP-9 concentration between individuals with the -1562CC genotype and the -1562CT genotype (smoking control: 385.67 ng/mL vs. 562.80 ng/mL, respectively, *p* = 0.000936; patients with other lung neoplasms: 821.64 ng/mL vs. 928.88 ng/mL, respectively *p* = 0.023315). The role of *MMP-2*-735C/T and *MMP-9* -1562C/T polymorphisms in an increased risk of lung cancer cannot be dismissed. Specific genotypes affect MMP-2 and MMP-9 concentrations in both lung cancer patients and healthy controls, which may thereby increase lung cancer risk, disease aggressiveness, and patient survival outcomes.

## 1. Introduction

Matrix metalloproteinases (MMPs) are a group of proteolytic enzymes which are capable of cleaving extracellular matrix (ECM) proteins. The first identified MMP was MMP-1, which was discovered in 1962 by Gross and Lapierre [1] as a result of collagen remodeling during tadpole tail metamorphosis. Since then, more than 28 MMPs have been discovered [2]. MMPs play a role in a variety of biological processes that occur during embryonic development, organogenesis, and wound healing, such as cell proliferation, migration, differentiation, tissue invasion, and vascularization. On the other hand, MMPs have piqued the interest of researchers due to their overexpression in numerous human disorders, including cardiovascular diseases, inflammatory diseases, lung and liver diseases, and malignancies, with lung cancer taking the lead [2,3,4,5].

Lung cancer accounts for nearly 25% of all cancer deaths worldwide. In 2020, an estimated 1,796,144 people died from lung cancer worldwide, which is not much fewer than the combined deaths from colorectal (935,173), breast (684,996), and prostate (375,304) cancer [6,7]. Such a high mortality rate of lung cancer patients is the effect, among others, of the fact that the vast majority of patients (75%) are diagnosed at an advanced stage of the disease, when treatment options are limited [8,9,10,11,12].

The leading cause of lung cancer development is tobacco use. Approximately 90% of lung cancer patients have a smoking history. However, only 10–20% (depending on the source of data) of people who smoke throughout their lives develop lung cancer [13,14,15]. Differences in these numbers imply that other factors, such as genetic susceptibility in the form of genotypic and phenotypic variables, including genetic polymorphism, contribute to lung carcinogenesis and influence individual differences in response to environmental factors, with carcinogens implicated in tobacco smoke exposure [9,13,16,17,18]. Over the last two decades, researchers have focused on low-penetrance genes involved in carcinogen metabolism and the DNA repair of damage caused by tobacco smoke, as well as changes in genes encoding proteins implicated in tumor formation, growth, and dissemination [19,20].

In the present study, we focused on low-penetrance variations in two genes of two MMPs, MMP-2 and MMP-9, which, along with MMP-1 and MMP-7, are the primary MMPs responsible for ECM remodeling in lung tissue [21,22]. MMP-2 and MMP-9 belong to gelatinases that, by degrading type IV collagen in the basement membrane, can contribute to carcinogenesis processes, such as cell proliferation, angiogenesis, and tumor metastasis when their activity is dysregulated [23,24,25]. MMP activity is regulated at several levels, including gene expression, compartmentalization, proenzyme activation, and enzyme inactivation [26,27,28].

The main studied polymorphisms of *MMP-2* are rs243865 and rs2285053, which are located in the *MMP-2* promoter at positions -1306 and -735, respectively, while the *MMP-9* polymorphism rs3918242 is located in the *MMP-9* promoter at position -1562, all of which induce the transition of the allele C to T [25]. *MMP-2* and *MMP-9* promoter polymorphisms can affect mRNA and protein expression levels by modifying transcriptional activity, eventually leading to the development of several types of cancer, including breast, lung, esophageal, and colorectal cancer [29,30,31,32]. According to growing research, *MMP-2* appears to also have a key role in the metastasis of a number of malignancies, including glioma and colorectal cancer [33,34].

In recent years, numerous genetic studies on *MMP-2* and *MMP-9*, along with their roles in cancer risk have been published. The majority of these studies, however, have been focused on breast, colorectal, or prostate cancer. In the previous decade, just five studies on the role of *MMP-2* and *MMP-9* polymorphism in lung cancer patients have been published and indexed in the MEDLINE (PubMed) database, which appears to be an understatement given the seriousness of the lung cancer problem. The objective of this study was to re-examine polymorphisms in the genes encoding the gelatinases MMP-2 and MMP-9 at positions -735C/T (rs2285053) and -1562C/T (rs3918242), respectively, and their potential effects on lung cancer, with our rationale for conducting this research being that our understanding of lung cancer has improved over the last decade. We sought answers to the following questions: (1) Can we identify populations at an increased risk of developing lung cancer by analyzing *MMP-2*-735C/T and *MMP-9*-1562C/T polymorphisms? (2) Do the polymorphisms *MMP-2*-735C/T and *MMP-9*-1562C/T affect the MMP-2 and MMP-9 concentrations? (3) Does the prevalence of certain polymorphic variants of *MMP-2* (735C/T) and *MMP-9* (1562C/T) vary amongst lung cancer subtypes?

## 2. Results

### 2.1. Characteristics of Cases and Controls

The study included 112 lung cancer patients and 100 healthy controls, including 47 non-smokers and 51 smokers, respectively. The selected characteristics of lung cancer patients and healthy individuals are summarized in Table 1. There were significant differences observed in the distribution of basic characteristics, such as age, gender, and smoking status between the cases and the controls. Lung cancer patients were mostly over the age of 60 (80.4% of patients), with an advantage of males (63.4%), and moderate (29.5%) to heavy (25.9%) smokers, whereas healthy individuals were mostly under the age of 60 (85.0% of controls), more often females (54.0%), and if smokers—light smokers (37.0%).

Table 2 presents basic information regarding the picked and analyzed SNPs, as well as the allele frequency distributions among the cases and controls. The Hardy–Weinberg equilibrium (HWE) was used to verify the observed *MMP-2*-735C/T and *MMP-9* -1562C/T genotype frequencies in the cases and controls (Table S1 of the Supplementary Materials). In the control group, we found a minor discrepancy in the frequency of the *MMP-2*-735C/T genotypes (*p* = 0.041271). When we subdivided the control group into non-smokers and smokers, we found a statistically significant difference between the observed and expected frequencies of the *MMP-2*-735C/T genotypes among non-smokers (*p* = 0.012686), but not among smokers (*p* = 0.600091).

In the analyses of both the *MMP-2*-735C/T and *MMP-9*-1562C/T polymorphisms, there were no statistically significant variations observed in the minor allele frequency (MAF) values between the patients and controls. Furthermore, when we investigated the relationship between the *MMP-2*-735C/T and *MMP-9*-1562C/T polymorphisms and ethnicity, we discovered that the MAFs received in our study’s population (0.1333 and 0.1659, respectively) correspond to MAFs that were obtained in other studies conducted on the European population (0.1101 and 0.1666, respectively).

We also conducted an analysis using the available data on the *MMP-2*-735C/T and *MMP-9*-1562C/T MAFs in various populations to examine whether there is any association between the ethnic distribution and the MMP mutation risk (Table 3). When we compared five major populations, i.e., European, East Asian, South Asian, American, and African, we found that the East Asian population (0.2591) had a statistically significantly higher T allele frequency of *MMP-2*-735C/T than the European (0.1101, *p* = 0.000134), South Asian (0.1454, *p* = 0.000132), African (0.1164, *p* = 0.000169), and American (0.1761, *p* = 0.000254) populations. Moreover, we observed statistically significantly higher MAF values in the American population compared to the European (*p* = 0.006904) and African (*p* = 0.008623) populations. There were no similar trends observed in the *MMP-9*-1562C/T T allele frequency. We observed the highest MAF values in the South Asian (0.2343) and African (0.2199) populations, and the lowest in the American (0.0809) population, with their *p*-values being at the limit of statistical significance of 0.053710 and 0.064237, respectively.

Next we investigated whether the *MMP-2*-735C/T and *MMP-9*-1562C/T genotypes were associated with environmental factors in lung cancer patients and healthy controls, as well as clinicopathological characteristics in lung cancer patients (Table 4). The only significant association we observed was between age and the *MMP-9*-1562C/T genotype in lung cancer patients (*p* = 0.03854), with lung cancer patients with the CT and TT genotypes being found to be more often younger than those with the CC genotype.

Of the total 112 lung cancer cases, 50 (44.6%) were adenocarcinoma, 35 (31.3%) were squamous cell carcinoma, and 27 (24.1%) were other lung neoplasms, respectively, including large-cell carcinoma, pleomorphic carcinoma, undifferentiated carcinomas, and metastases of other neoplasms to the lungs. Table 5 shows the selected characteristics of lung cancer patients divided by their lung tumor subtype. Lung cancer patients did not significantly differ between their subtypes in terms of their age, gender, and smoking status. There were also no associations found between the genotypes of *MMP-2*-735C/T and *MMP-9*-1562C/T and the lung cancer subtype (Table 4). However, there were significant differences observed in the frequency of metastases between adenocarcinoma patients and patients with other lung neoplasms (*p* = 0.01420), as well as between squamous cell carcinoma patients and patients with other lung neoplasms (*p* = 0.00701), with squamous cell carcinoma patients having the highest frequency of metastases to the lymph nodes and patients with other lung neoplasms having the highest frequency of distant metastases. Despite the metastasis factor, the characteristics of the lung cancer patient group by lung tumor subtype were found to be homogeneous, which was important information for further analyses.

### 2.2. The Effect of Dependent Variables on the Risk of Developing Lung Cancer

Prior to examining the impact of these specific *MMP-2*-735C/T and *MMP-9*-1562C/T genotypes on the MMP-2 and MMP-9 concentrations, we performed logistic regression analyses to estimate the impact of each examined factor on the risk of lung cancer. Table 6 summarizes the results of logistic regression analyses with comparisons between the controls and lung cancer patients. We observed that male gender increased the risk of lung cancer by two-fold. Aging and an increase in the number of smoked pack-years were also found to have statistically significantly increased the risk of lung cancer development. With every year, the risk of lung cancer incidence increased by 23%, whereas each packyear smoked increased the risk of lung cancer by 15%, respectively. Logistic regression analysis confirmed the Pearson’s chi-square test results in terms of the significance of the *MMP-2*-735C/T and *MMP-9*-1562C/T genotype frequencies between the lung cancer patients and the controls. Despite the lack of statistical significance, we observed that the *MMP-2*-735CC genotype increases the lung cancer risk by 5-fold, while the CT genotype increases the risk by 7-fold, respectively. In case of the *MMP-9*-1562C/T genotypes the increase was not as significant, but it was still 45% and 60% for the CC and the CT genotypes, respectively. Importantly, we observed that both the decrease in the MMP-2 concentration and the increase in the MMP-9 concentration further enhance the risk of lung cancer development.

### 2.3. Concentration of MMP-2 Depending on the MMP-2-735C/T Genotypes

MMP-2 concentrations were compared in two groups of controls, non-smokers (NSC) and smokers (SC), with three lung cancer subtypes, including adenocarcinoma (ADC), squamous cell carcinoma (SqCC), and other lung neoplasms (OLN), in relation to the *MMP-2*-735C/T genotype. There were no significant differences observed in the MMP-2 concentrations between the non-smokers and smokers in both the -735CC and -735CT genotypes. However, there were statistically significant differences observed in the MMP-2 concentration among the non-smoking controls with the -735CC genotype ($\bar{x}$ = 204.04 ng/mL) and the -735CT genotype ($\bar{x}$ = 237.00 ng/mL, *p* = 0.041479).

**Figure 1:**
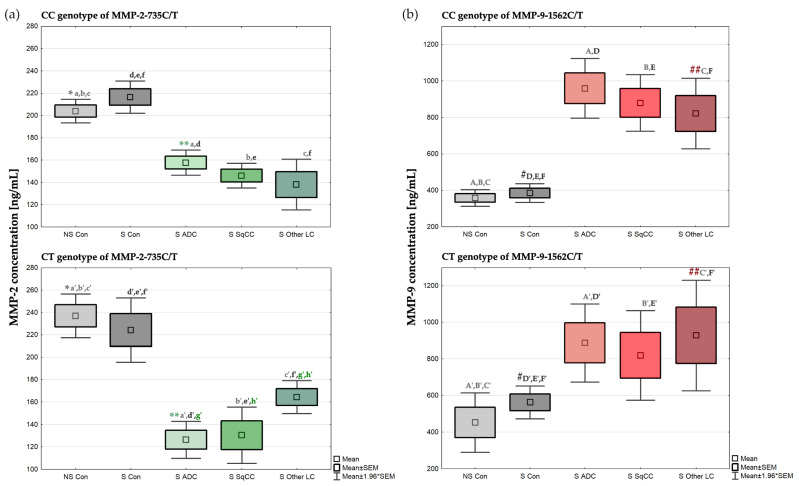
Comparisons of the (**a**) MMP-2 concentrations by the MMP-2-735C/T genotype and (**b**) the MMP-9 concentrations by the MMP-9-1562C/T genotype observed in the lung cancer cases and controls. Controls are depicted in grey in both the MMP-2 and MMP-9 concentration analyses, with non-smoking controls being lighter and smoking controls being darker. MMP-2 concentrations in cases are depicted in green color, with the lightest green in adenocarcinoma patients, medium green in squamous cell carcinoma patients, and darkest green in patients with other lung neoplasms. Similarly, MMP-9 concentrations in cases are depicted in red color, with the lightest red in adenocarcinoma patients, medium red in squamous cell carcinoma, and darkest red in patients with other lung neoplasms. All statistical significances are detailed in the notes section of Table 7.

Within both of the CC and CT genotypes of the *MMP-2*-735C/T polymorphism, patients with all lung cancer subtypes (ADC, SqCC, and OLN) had statistically significant differences in their MMP-2 concentrations, with lower levels of MMP-2 observed compared to the non-smoking and smoking controls. Within the -735CC genotype, we observed the highest MMP-2 concentration in the smoking control ($\bar{x}$ = 216.56 ng/mL) and the lowest in patients with other lung neoplasms ($\bar{x}$ = 138.05 ng/mL). In the -735CT genotype, we found the highest MMP-2 level in the non-smoking control ($\bar{x}$ = 237.00 ng/mL), and the lowest in adenocarcinoma patients ($\bar{x}$ = 126.37 ng/mL). All statistical significances are detailed in the notes section of Table 7, and these results are also presented in Figure 1a.

Furthermore, the MMP-2 concentration was found to be statistically significantly higher in adenocarcinoma patients with the -735CC genotype ($\bar{x}$ = 157.69 ng/mL) than in adenocarcinoma patients with the -735CT genotype ($\bar{x}$ = 126.37 ng/mL, *p* = 0.013222). In contrast, we observed lower concentrations of MMP-2 in patients with other lung neoplasms and the -735CC genotype ($\bar{x}$ = 138.05 ng/mL), than in patients with other lung neoplasms and the -735CT genotype ($\bar{x}$ = 164.48 ng/mL) at the limit of statistical significance (*p* = 0.060294). In patients with other lung neoplasms and the -735CT genotype, MMP-2 concentrations were also found to be significantly higher than in patients with the -735CT genotype and adenocarcinoma ($\bar{x}$ = 164.48 ng/mL vs. $\bar{x}$ = 126.37 ng/mL, respectively, *p* = 0.003789), and squamous cell carcinoma ($\bar{x}$ = 164.48 ng/mL vs. $\bar{x}$ = 130.43 ng/mL, respectively, *p* = 0.029928). Table 7 summarizes all MMP-2 concentration results by the patient group and genotype of the *MMP-2*-735C/T polymorphism.

### 2.4. Concentration of MMP-9 Depending on the MMP-9-1562C/T Genotypes

We used the same patient classification and calculation method for MMP-9 concentration analysis as we performed for MMP-2. Similarly, there was no significant difference observed in the MMP-9 concentration between the non-smokers and smokers of the control group in both the -1562CC and -1562CT genotypes. However, a statistically significant difference in the MMP-9 concentration was found between the smoking controls with the -1562CC genotype ($\bar{x}$ = 385.67 ng/mL) and smoking controls with the -1562CT genotype ($\bar{x}$ = 562.80 ng/mL, *p* = 0.000936).

Patients with all lung cancer subtypes (ADC, SqCC, and OLN) were found to have statistically significant differences in their MMP-9 concentrations with higher levels of MMP-9 observed compared to the non-smoking and smoking controls within both the CC and CT genotypes of the *MMP-9*-1562C/T polymorphism. Within the -1562CC genotype, we observed the highest MMP-9 concentration in the adenocarcinoma patients ($\bar{x}$ = 959.95 ng/mL) and the lowest in the non-smoking controls ($\bar{x}$ = 358.74 ng/mL). Furthermore, we found the highest MMP-9 concentration in the -1562CT genotype in patients with other lung neoplasms ($\bar{x}$ = 928.88 ng/mL), and the lowest was once again observed in the non-smoking group ($\bar{x}$ = 452.62 ng/mL). All statistical significances are detailed in the notes section of Table 7, and these results are also presented in Figure 1b.

In contrast to the MMP-2 concentration, there were no statistically significant differences observed in the MMP-9 concentration between the lung cancer subtypes within both the -1562CC and -1562CT genotypes. However, we observed that patients with other lung neoplasms with the -1562CT genotype had a statistically higher MMP-9 concentration ($\bar{x}$ = 928.88 ng/mL) than patients with other lung neoplasms with the -1562CC genotype ($\bar{x}$ = 821.64 ng/mL, *p* = 0.023315). Table 7 summarizes all MMP-9 concentration results by the patient group and *MMP-9*-1562C/T polymorphism genotype.

## 3. Discussion

Even though tobacco smoke exposure causes lung cancer in 90% of cases, each patient may have a unique molecular pattern that causes this disease. The mechanisms by which smoking promotes lung carcinogenesis are numerous in quantity [35,36,37,38]. Individual vulnerability to tobacco smoke, also known as genetic susceptibility, can occur as a result of (1) the inheritance of low-frequency, high-penetrance genes; (2) the inheritance of high-frequency, low-penetrance genes; or (3) acquisition via epigenetic mechanisms. Candidates for lung cancer susceptibility genes have been intensively researched, with an emphasis on the variation in predisposing, low-penetrance genes involved in carcinogen metabolism and the DNA repair of damage caused by tobacco smoke, as well as changes in the genes encoding proteins implicated in tumor formation, growth, and dissemination [19,20]. In this study, we evaluated the effect of two polymorphisms in the promoter regions of two human gelatinases, i.e., MMP-2 and MMP-9, on the risk of lung cancer development.

Among the secreted MMPs, MMP-2 and MMP-9 have long been considered to play an important role in cancer invasion and metastasis due to their ability to degrade the ECM and basement membrane barriers required for each step of tumor progression [39,40,41,42,43]. Recent studies have, however, demonstrated that MMP functions are much more complex, since they are the key mediators of growth factor activation, bioavailability, receptor signaling, cell adhesion and motility, apoptosis and survival mechanisms, angiogenesis, inflammatory responses, and immunological surveillance [44]. As a result, polymorphisms in these MMP genes are being extensively studied in patients suffering from various malignancies. However, the number of published genotypic articles on the role of MMP-2 and MMP-9 in lung cancer has been extremely low in the preceding decade.

We analyzed two SNPs with known functional effects, i.e., rs2285053 of the *MMP-2* gene at position -735 with a C-to-T transition that has been shown to destroy the binding site of specificity protein 1 (Sp1) to MMP-2 mRNA, resulting in the reduction of its transcription level; and rs3918242 of the *MMP-9* gene at position -1562, also with a C-to-T transition resulting in the change in the promoter’s activity [29,45,46]. SNPs were analyzed in 112 lung cancer patients and 100 healthy controls in association with the MMP-2 and MMP-9 serum concentrations, revealing that specific genotypes appeared to affect both the MMP-2 and MMP-9 concentrations, which may result in an increased lung cancer risk, a more aggressive course of the disease, and poorer patient survival outcomes.

In our study, the *MMP-2*-735C/T genotype frequencies were found to be CC 74.1%, CT 23.2%, and TT 0.9% in the lung cancer patients group, and CC 77.0%, CT 18.0%, and TT 5.0% in the control group, respectively. There were no statistically significant differences observed in the distribution of the *MMP-2*-735C/T genotypes. As expected, there were no statistically significant differences observed in the MAF of the rs2285053 values between the cases (0.1273) and the controls (0.1400). However, the distribution of the *MMP-2* genotypes in the controls in our study was not in HWE. Interestingly, Gonzalez-Arriaga et al. (2012) [44] observed the same issue in their analysis, which was also based on the European population. Similarly, to Gonzalez-Arriaga et al., the explanation for this problem in our case is unknown, as we used a control group of healthy individuals that were randomly recruited and received the consistency with the HWE in other polymorphic loci—in the *MMP-9* gene located at the -1562 position.

According to logistic regression analysis using the -735TT genotype as the reference level, the prevalence of the -735CC genotype was found to be associated with a 5.4-fold higher risk of developing lung cancer, whereas the -735CT genotype was associated with a 7.2-fold higher risk, respectively. Even though the results were not statistically significant (*p*-values 0.238504 and 0.072836, respectively), the odds ratios remained quite high, which may be due to the fact that there were no patients with the -735TT genotype among our research group’s adenocarcinoma and squamous cell carcinoma patients. On the one hand, the lack of the presence of the -735TT genotype among patients with adenocarcinoma and squamous cell carcinoma may weight our results with an error, but on the other hand, it may be a normal trend among the two most common subtypes of lung cancer. Individuals with the TT genotype of the *MMP-2*-735C/T polymorphism had a lower risk of lung cancer when compared to the CC genotype, according to Wang et al. (2012) [43], and Li et al. (2015) [24], and the TT genotype had a protective effect as a result of a lower promoter activity, and thus lowered the MMP-2 enzyme activity. In our study, a larger sample size would have revealed more about the importance of these findings.

The genotype frequencies for the *MMP-9*-1562C/T polymorphism in lung cancer patients were CC 67.9%, CT 26.8%, and TT 2.7%, and CC 70.0%, CT 25.0%, and TT 4.0% in the control group, respectively, with no statistically significant differences observed between these two groups. In addition, no statistically significant differences in the MAF of the rs3918242 values were found between the cases (0.1651) and controls (0.1667). A few studies have indicated that individuals carrying the T allele had a lower risk of developing lung cancer, whereas those carrying the C allele had an increased risk [24,42,44]. In our study, logistic regression analysis revealed a 1.4-fold increased risk of developing lung cancer in carriers of the -1562CC genotype, and a 1.6-fold increased risk in carriers of the -1562CT genotype, respectively, although these results were not statistically significant. The findings of Bayramoglu et al. (2009) [39] and Rollin et al. (2007) [45] were consistent with the findings obtained in this study, indicating that there is no difference in the distribution of the *MMP-9*-1562C/T genotypes between the lung cancer patients and healthy individuals. Furthermore, Wang et al. (2005) [46] suggested that rs3918242 may not be a good marker for predicting lung cancer susceptibility and the presence of lymphatic metastasis in lung cancer patients.

The reported results of the *MMP-2*-735C/T and *MMP-9*-1562C/T polymorphisms and their role in lung cancer risk are frequently conflicting. The source of the contradictory results could be explained by their ethnicity, producing variation as a result of their genetic backgrounds as well as environmental factors across the different ethnicities. For example, the study by Li et al. (2015) [24] produced significant results of the *MMP-2*-735C/T polymorphism in the overall comparison and among Asians, but not among the Caucasians, whereas the *MMP-9*-1562C/T polymorphism was solely significant among the Asians. To assess the significance of our results in the Polish population, we compared them to the available genomic data on rs2285053 and rs3918242 from projects, such as 1000Genomes, 1000Genomes_30x, Allele Frequency Aggregator, gnomAD-Genomes, and the PAGE Study. We found that the T allele frequencies (minor allele frequencies) of both the *MMP-2*-735C/T and *MMP-9*-1562C/T polymorphisms in our study population (0.1333, and 1659, respectively) were consistent with the average MAF values in the European population (0.1101 and 0.1666, respectively). When we compared the *MMP-2*-735C/T polymorphism in five major populations, including the European, East Asian, South Asian, American, and African populations, we found statistically significant differences, with the East Asian population having the highest MAF value (0.2591), and the European population having the lowest (0.1101). The *MMP-9*-1562C/T, on the other hand, revealed no statistically significant differences in the MAF values between the ethnicities, with the South Asian population having the highest MAF value (0.2343), and the American population having the lowest (0.0809). The findings corroborate the association between ethnicity and the MMP mutation risk and explain the disparities in the results of studies conducted on different ethnicities. It further demonstrates that ethnicity must be taken into account when assessing the risk of developing lung cancer based on the *MMP* polymorphisms.

We also investigated whether the *MMP-2*-735C/T and *MMP-9*-1562C/T genotypes affected the MMP-2 and MMP-9 concentration levels. We observed significant differences in the MMP-2 and MMP-9 concentrations between the -735CC and -735CT genotypes, as well as the -1562CC and -1562CT genotypes, respectively, not only among the lung cancer subtypes but also among the healthy non-smokers and smokers. We found statistically significant higher MMP-2 concentrations in non-smokers with the -735CT genotype and higher MMP-9 concentrations in smokers with the -1562CT genotype. Moreover, we observed an opposite effect in lung cancer patients with adenocarcinoma, the squamous cell carcinoma subtypes, and the -735CC genotype which all had statistically increased MMP-2 levels, as well as patients with the -1562CC genotype who had higher MMP-9 concentration levels than in patients with the -1562CT genotype. These findings confirm that polymorphisms at the promoter regions of *MMPs* affect the expression levels of these proteins.

**Figure 2:**
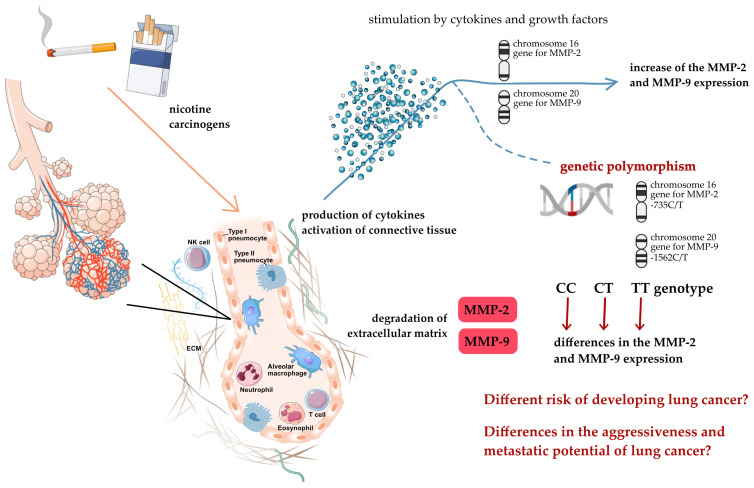
Mechanisms of MMP-2 and MMP-9 stimulation by tobacco smoke exposure. Created with BioRender.com and Affinity Designer. When exposed to tobacco smoke, pulmonary alveoli are stimulated to release cytokines by both epithelial and inflammatory cells; increased cytokine production then causes an inflow of inflammatory cells into the alveoli, exacerbating local inflammation, as well as activation of the connective tissue, i.e., ECM, with the stimulation of the *MMP-2* and *MMP-9* genes in cells producing these MMPs, resulting in an increase in MMP expression; carriers of genotypes predisposed to elevated MMP concentrations are at an increased risk of being transformed or preinvasive lung cells caused by tobacco carcinogens being converted into an invasive tumor under the conditions of a higher lifetime MMP expression.

Several articles have reported that MMP expression can be induced by smoking, leading changes in the MMP/TIMP ratio [47]. The effect of cigarette smoking on MMP concentrations was also found in our research. We observed the increase in the MMP-9 concentration in the smoking controls when compared to the non-smoking controls. Even though the increase was not statistically significant, we noticed a trend since smoking controls for each *MMP-9*-1562C/T genotype had higher MMP-9 concentrations than the non-smoking controls for each genotype (385.67 ng/mL, 562.80 ng/mL, and 648.57 ng/mL vs. 312.41 ng/mL, 452.62 ng/mL, and 358.70 ng/mL for the CC, CT, and TT genotypes of the smoking and non-smoking control groups, respectively). The increase in the MMP levels in the smoking controls (as compared to the non-smoking controls) suggests that smoking is the factor that induces ECM remodeling. Moreover, connective tissue remodeling may promote tumor development [22,41,43]. As the great majority of lung cancer patients have a smoking history and tobacco-smoke-related increased MMP expression, the subsequent ECM remodeling that arises as a result may be regarded as one of the initial steps of lung carcinogenesis (Figure 2).

Moreover, there have been reports published regarding the additive joint effect of smoking and *MMP* genotypes on lung cancer risk determination. Zhou et al. (2005) [46] investigated the synergistic effect of two polymorphism in the *MMP-2* promoter region at the locations -735 and -1306 with a C-to-T transition. The authors found that the C-735-C-1306 haplotype was associated with an increased risk of lung cancer when compared to the T-735-T-1306 haplotype, and the risk of developing lung cancer being even higher in smokers with the C-735-C-1306 haplotype. At this point, it could be expected that individuals who smoke and carry the *MMP-2*-735CC or -1306CC genotype, or the C-735-C-1306 haplotype, are more susceptible to develop lung cancer than those who smoke and carry either the -735TT or -1306TT genotype, or the T-735-T-1306 haplotype.

In our study, we also observed greater levels of MMP-2 in patients with other lung neoplasms than in patients with adenocarcinoma or squamous cell carcinoma that could be attributed to a higher prevalence of distant metastases in this group of patients, which included individuals with metastases to the lungs from other neoplasms. The group of patients with other lung neoplasms was characterized by statistically significant differences in the metastases factor when compared with both adenocarcinoma (22.2% vs. 8.0%, respectively, *p* = 0.01420) and squamous cell carcinoma patients (22.2% vs. 5.7%, respectively, *p* = 0.00701). These observations may confirm a role of MMP-2 in cancer metastasis and its higher serum expression due to metastasis. Since a higher MMP-2 level has been associated with an increased risk of lung cancer, a more aggressive course of the disease, and a higher incidence of distant metastases [23,48,49], we can thereby predict that lung cancer patients with the -735CC genotype, which predisposes to higher MMP concentrations, will have a worsened prognosis and shorter overall survival times than patients with the CT or TT genotype. Gonzalez-Arriaga et al. (2012) [44] partially confirmed this hypothesis by demonstrating that the *MMP-2*-735C allele was related with shorter survival times in carriers compared to those carrying the T allele (*p* = 0.02).

The comparison of lung cancer subtypes in the prevalence of *MMP-2*-735C/T and *MMP-9*-1562C/T genotypes complemented our research. There were no statistically significant changes observed in the frequency of the *MMP-2*-735C/T and *MMP-9*-1562C/T genotypes between patients with adenocarcinoma, squamous cell carcinoma, and other lung neoplasms. We also found no significant differences in the frequency of the *MMP-2*-735C/T and *MMP-9*-1562C/T genotypes between lung cancer patients with no metastases, lymph nodes metastases, and distant metastases. There were also no variations observed in the MMP-2 and MMP-9 concentrations based on metastasis incidence. However, we cannot rule out the impact of *MMP* polymorphism on the lung cancer subtype and metastasis presence, since our above-described analyses demonstrate differences in the MMP-2 and MMP-9 concentrations between these lung cancer subtypes based on the *MMP-2*-735C/T and *MMP-9*-1562C/T genotypes, respectively.

Cancer is a multifactorial disease that results from complex interactions between the hereditary and environmental factors. Lung cancer is an aggressive and genomically unstable cancer that progresses with a series of genetic and epigenetic changes [38,50]. It may be naive to expect that a single mutation or nucleotide variation predominates the progression of cancer. Although the differences in allele transcription caused by polymorphisms in the *MMP* promoters are subtle when compared to, for example, oncogene overexpression [46,51], specific genotypes have appeared to affect the concentrations of MMP-2 and MMP-9, which when increased may result over a lifetime in an increased susceptibility to lung cancer, a more aggressive course of the disease, and poorer patient survival outcomes. Candidates for single nucleotide polymorphisms implicated in increased lung cancer risk must be sought for in this regard in order to better understand and link the individual factors involved in lung cancer pathogenesis, and thus improve the diagnostic, screening, and therapeutic options for future lung cancer patients.

## 4. Materials and Methods

### 4.1. Patients

We present a non-interventional, retrospective, case-control study. The case group comprised 112 lung cancer patients recruited by the Department of Thoracic Surgery, Lower Silesian Centre for Lung Diseases in Wroclaw, Poland. All participants signed a written informed consent following an explanation of the study protocols. The study protocol conformed to the World Medical Association’s Declaration of Helsinki (2000) and was approved by the Bioethics Committee at the Wroclaw Medical University (NR KB: 106/2020 and 433/2022). Clinical, laboratory, and pathological data for these patients were acquired from hospital medical records using the AMMS IT system (Asseco Medical Management Solutions). Lung cancer diagnosis was established in accordance with the National Comprehensive Cancer Network (NCCN) Clinical Practice Guidelines in Oncology and was staged in accordance with the American Joint Committee on Cancer’s (AJCC) 8th TNM Staging System.

The control group comprises 100 volunteers, 47 of whom were healthy non-smokers and 51 of whom were healthy smokers, respectively. The Biobank Research Group, Łukasiewicz Research Network—PORT Polish Centre for Technology Development provided biological material (sera, whole blood for DNA isolation) and basic data about patients in the control group to the Department of Medical Laboratory Diagnostics, Division of Clinical Chemistry, and Laboratory Hematology. All provided samples were stored at −80 °C until at the point of their utilization.

### 4.2. Methods

Following admission to the hospital, venous blood samples were collected into tubes with ethylenediaminetetraacetic acid (EDTA) anticoagulant and tubes with the clot activator from all lung cancer patients. Then, 200 μL of whole blood was taken from EDTA blood samples for DNA isolation. Following this, at room temperature, blood samples were centrifuged at 2000× *g* for 8–10 min to separate the plasma and serum, which were then stored at −80 °C until their use.

Patients’ exposure to cigarette smoke was assessed based on their smoking history and nicotine metabolite (cotinine) concentrations in their sera. Participants were divided into two groups based on their tobacco consumption: those who had never smoked, defined as subjects who had not smoked at least one cigarette per day regularly for six months or longer in their lifetimes, and those who smoked, including former smokers, who were defined as regular smokers who had quit smoking at least one year before the interview; and current smokers, who were defined as subjects who are active smokers. The concentration of cotinine was measured to confirm active smoking. Cotinine serum levels were determined using a competitive immunoenzymatic assay termed Cotinine direct (Serum/Urine) ELISA (Cat. No: EIA-5496/EIA-5497, DRG International Inc., Springfield, NJ, USA).

Serum blood samples were also used to determine the concentrations of MMP-2 and MMP-9. Commercial enzyme-linked immunosorbent assays (ELISA) test kits: Total MMP-2 Quantikine ELISA (Catalog # MMP200), and Human MMP-9 Quantikine ELISA (Catalog # DMP900), R&D Systems, Inc., Minnesota, MN, USA, were used in accordance with the manufacturer’s protocols. ELISA kits characteristics are presented in Table 8.

DNA isolation was performed using the binding column technology of the Syngen Blood/Cell DNA Mini Kit (300) (Cat. No: SY221012, Syngen, Poland). The NanoDrop™ Lite spectrophotometer (Thermo Fisher Scientific, Waltham, MA, USA) was used to quantify the purity (the A260/A280 ratio) and concentration (the A260 measurement) of the isolated DNA. All extractions had a high purity of ~1.8, along with an average yield of DNA of 14.55 μg in the case group and 10.77 μg in the control group, respectively (a typical DNA extraction yield from frozen whole blood samples using this kit is between 4 and 12 μg, respectively). Isolated DNAs were stored at −80 °C until their use.

**Figure 3:**
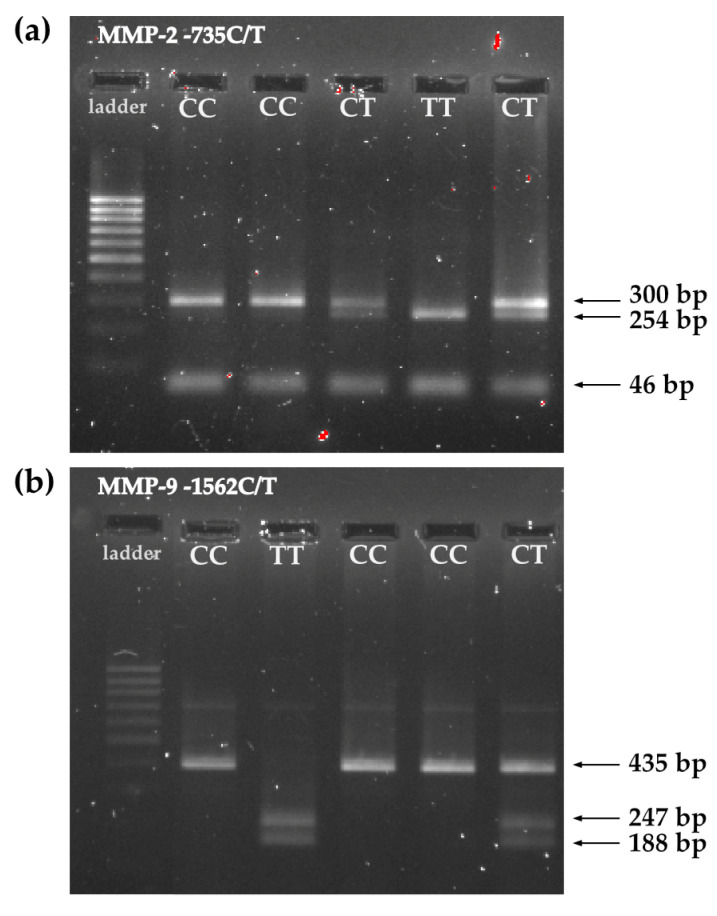
Examples of the electrophoretic separation of the PCR-RFLP products from the (**a**) *MMP-2* promoter -735C/T polymorphism analysis; and the (**b**) *MMP-9* promoter -1562C/T polymorphism analysis.

The polymerase chain reaction method with restriction fragment length polymorphism (PCR/RFLP) was used for genotyping. The PCR-RFLP method consists of three analysis steps. The promoter regions containing polymorphisms were amplified using PCR to obtain the amount of DNA needed for RFLP analysis. Then, the amplified DNA sequences were cut with specific restriction endonucleases. Finally, after digestion of the DNA samples, DNA fragments of various lengths were separated using gel electrophoresis in 1.5% agarose gel and analyzed under UV light to reveal differences in the homologous DNA sequences. Table 9 contains a detailed protocol for the PCR-RFLP method, while Table 10 contains the primer sequences. Figure 3 shows a photo of the electrophoretic separation of (a) *MMP-2*-735C/T polymorphism analysis products and (b) *MMP-9*-1562C/T polymorphism analysis products. We performed both random duplications in 20% of the samples to reduce the genotyping error rate.

In our study, we also used results from the *MMP-2*-735C/T and *MMP-9*-1562C/T MAF (in both cases, T allele frequency) available on the website of the Reference SNP (rs) Report of National Library of Medicine, National Centre for Biotechnology Information (https://www.ncbi.nlm.nih.gov/snp/rs2285053, https://www.ncbi.nlm.nih.gov/snp/rs3918242, respectively, both accessed on 3 June 2023).

### 4.3. Statistical Analysis

The obtained data were statistically analyzed using TIBCO Software Inc. (Palo Alto, CA, USA) (2017), Statistica, version 13 (http://statistica.io, accessed on 17 December 2022) with the additional Plus Package (version 5.0.96), and a significance level of *p* < 0.05. The Shapiro–Wilk test was used to determine whether the data for each parameter was normally distributed across all analyzed groups. Pearson’s chi-square test was then applied to analyze sets of categorical data, including deviation from the HWE. Logistic regression analysis was also used to estimate qualitative data. To compare independent, continuous variables between two groups, the parametric Student’s *t*-test or the non-parametric Mann–Whitney U test were used, and the one-way analysis of variance (ANOVA) was used if there were more groups. Post-hoc analyses using Tukey’s Honest Significant Difference (HSD) corrected for unequal sample sizes supplemented the ANOVA.

## Figures and Tables

**Table 1:** Selected characteristics of the lung cancer patients and controls.

Variable	Cases [*n*, (%)] *n* = 112	Controls [*n*, (%)] *n* = 100	*p*-Value (Pearson’s Chi-Square Test)
Age [years]			
≤60	22 (19.6%)	85 (85.0%)	*p* < 0.00001
>60	90 (80.4%)	15 (15.0%)	
Gender			
Male	71 (63.4%)	46 (46.0%)	*p* = 0.01102
Female	41 (36.6%)	54 (54.0%)	
Smoking status			
Never smoker	3 (2.7%)	47 (47.0%)	*p* < 0.00001
Light smoker	7 (6.3%)	37 (37.0%)	
Moderate smoker	33 (29.5%)	11 (11.0%)	
Heavy smoker	29 (25.9%)	2 (2.0%)	
NA	40 (35.7%)	3 (3.0%)	
*MMP-2*-735 C/T			
CC	83 (74.1%)	77 (77.0%)	*p* = 0.14379
CT	26 (23.2%)	18 (18.0%)	
TT	1 (0.9%)	5 (5.0%)	
NA	2 (1.8%)	0 (0.0%)	
*MMP-9*-1562 C/T			
CC	76 (67.9%)	70 (70.0%)	*p* = 0.83358
CT	30 (26.8%)	25 (25.0%)	
TT	3 (2.7%)	4 (4.0%)	
NA	3 (2.7%)	1 (1.0%)	

Notes: Light smokers are defined as people who smoke from >0 to <20 pack-years; moderate smokers are defined as people who smoke from ≥20 to <40 pack-years; and heavy smokers are defined as people who smoke ≥40 pack-years. NA—not available.

**Table 2:** Characteristics of the analyzed single nucleotide variations.

SNP	Gene	Band	Position	Alleles	Molecular Consequences	MAF—Cases	MAF—Controls	*p*-Value
rs2285053	*MMP-2*	16q12.2	55478465	C>T	2KB upstream variant	0.1273	0.1400	0.7376
rs3918242	*MMP-9*	20q13.2	46007337	C>T	2KB upstream variant	0.1651	0.1667	0.9717

**Table 3:** Descriptive statistics on the minor allele frequencies of rs2285053 and rs3918242 across various populations.

Population	N	Mean ± SEM	Median	Min–Max
*MMP-2*-735C/T (rs2285053)				
European	9	0.1101 ± 0.0087 **^a,b^**	0.1133	0.0500–0.1346
East Asian	9	0.2591 ± 0.0047 **^a,c,d,e^**	0.2604	0.2368–0.2784
South Asian	5	0.1454 ± 0.0280 **^c^**	0.1186	0.1110–0.2570
African	5	0.1164 ± 0.0020 **^d,f^**	0.1153	0.1105–0.1221
American	3	0.1761 ± 0.0048 **^b,e,f^**	0.1801	0.1666–0.1816
Semitic	2	0.1411 ± 0.0024	0.1411	0.1387–0.1435
Latin 1	2	0.1227 ± 0.0132	0.1227	0.1096–0.1358
Latin 2	2	0.1872 ± 0.0068	0.1872	0.1803–0.1940
Other	2	0.1393 ± 0.0102	0.1393	0.1291–0.1494
*MMP-9*-1562C/T (rs3918242)				
European	8	0.1666 ± 0.0051	0.1671	0.1463–0.1873
East Asian	8	0.1583 ± 0.0110	0.1562	0.1294–0.2260
South Asian	4	0.2343 ± 0.0415 *	0.2427	0.1250–0.3269
African	5	0.2199 ± 0.0788 **	0.1214	0.1103–0.5231
American	3	0.0809 ± 0.0039 *,**	0.0796	0.0749–0.0883
Semitic	2	0.1610 ± 0.0057	0.1610	0.1553–0.1667
Latin 1	1	0.1096	0.1096	
Latin 2	1	0.0836	0.0836	
Other	2	0.1741	0.1741	0.1257–0.2225

Notes: N—number of studies included; SEM—standard error of the mean; statistical significances, one-way ANOVA: ^a^ *p* = 0.000134 between Europeans and East Asians, ^b^ *p* = 0.006904 between Europeans and Americans, ^c^ *p* = 0.000132 between East Asians and South Asians, ^d^ *p* = 0.000169 between East Asians and Africans, ^e^ *p* = 0.000254 between East Asians and Americans, and ^f^ *p* = 0.008623 between Africans and Americans in the rs2285053 MAF; * *p* = 0.053710 between South Asians and Americans, and ** *p* = 0.064237 between Africans and Americans in the rs3918242 MAF.

**Table 4:** Association of the (**a**) rs2285053 and (**b**) rs3918242 genotypes with selected qualitative variables in lung cancer patients and in healthy controls.

**(a)** ***MMP-2*-735C/T (rs2285053)**							
**Variable**	**Cases**			**Controls**			***p*-Value (Pearson’s Chi-Square Test)**
	**CC (*n* = 83)**	**CT (*n* = 26)**	**TT (*n* = 1)**	**CC (*n* = 77)**	**CT (*n* = 18)**	**TT (*n* = 5)**	
Age [years]							
≤60	18 (21.7%)	3 (11.5%)	1 (100.0%)	65 (84.4%)	16 (88.9%)	4 (80.0%)	^a^ *p* = 0.07026
>60	65 (78.3%)	23 (88.5%)	0 (0.0%)	12 (15.6%)	2 (11.1%)	1 (20.0%)	^b^ *p* = 0.84697
Gender							
Male	54 (65.1%)	15 (57.7%)	1 (100.0%)	35 (45.5%)	9 (50.0%)	2 (40.0%)	^a^ *p* = 0.59419
Female	29 (34.9%)	11 (42.3%)	0 (0.0%)	42 (54.5%)	9 (50.0%)	3 (60.0%)	^b^ *p* = 0.90591
Smoking status							
Never smoker	2 (2.4%)	1 (3.8%)	0 (0.0%)	39 (50.6%)	5 (27.8%)	3 (60.0%)	^a^ *p* = 0.90223
Light smoker	7 (8.4%)	0 (0.0%)	0 (0.0%)	27 (35.1%)	8 (44.4%)	2 (40.0%)	^b^ *p* = 0.45752
Moderate smoker	25 (30.1%)	7 (26.9%)	0 (0.0%)	7 (9.1%)	4 (22.2%)	0 (0.0%)	
Heavy smoker	23 (27.7%)	6 (23.1%)	0 (0.0%)	2 (2.6%)	0 (0.0%)	0 (0.0%)	
NA	26 (31.3%)	12 (46.2%)	1 (100.0%)	2 (2.6%)	1 (5.6%)	0 (0.0%)	
Lung cancer subtype							
Adenocarcinoma	39 (47.0%)	10 (38.5%)	0 (0.0%)				^a^ *p* = 0.21120
Squamous cell carcinoma	28 (33.7%)	7 (26.9%)	0 (0.0%)				
Other lung neoplasms	16 (19.3%)	9 (34.6%)	1 (100.0%)				
Metastases							
No metastases	40 (48.2%)	11 (42.3%)	1 (100.0%)				^a^ *p* = 0.80839
To lymph nodes	32 (38.6%)	12 (46.2%)	0 (0.0%)				
Distant metastases	11 (13.3%)	3 (11.5%)	0 (0.0%)				
**(b)** ***MMP-9*-1562C/T (rs3918242)**							
**Variable**	**Cases**			**Controls**			***p*-Value (Pearson’s Chi-Square Test)**
	**CC (*n* = 76)**	**CT (*n* = 30)**	**TT (*n* = 3)**	**CC (*n* = 70)**	**CT (*n* = 25)**	**TT (*n* = 4)**	
Age [years]							
≤60	11 (14.5%)	8 (26.7%)	2 (66.7%)	61 (87.1%)	20 (80.0%)	3 (75.0%)	^a^ *p* = 0.03854
>60	65 (85.5%)	22 (73.3%)	1 (33.3%)	9 (12.9%)	5 (20.0%)	1 (25.0%)	^b^ *p* = 0.59287
Gender							
Male	47 (61.8%)	20 (66.7%)	3 (100.0%)	30 (42.9%)	13 (52.0%)	3 (75.0%)	^a^ *p* = 0.37972
Female	29 (38.2%)	10 (33.3%)	0 (0.0%)	40 (57.1%)	12 (48.0%)	1 (25.0%)	^b^ *p* = 0.37092
Smoking status							
Never smoker	2 (2.6%)	1 (3.3%)	0 (0.0%)	35 (50.0%)	10 (40.0%)	2 (50.0%)	^a^ *p* = 0.84560
Light smoker	6 (7.9%)	1 (3.3%)	0 (0.0%)	25 (35.7%)	10 (40.0%)	1 (25.0%)	^b^ *p* = 0.91162
Moderate smoker	25 (32.9%)	7 (23.3%)	0 (0.0%)	7 (10.0%)	3 (12.0%)	1 (25.0%)	
Heavy smoker	18 (23.7%)	11 (36.7%)	0 (0.0%)	1 (1.4%)	1 (4.0%)	0 (0.0%)	
NA	25 (32.9%)	10 (33.3%)	3 (100.0%)	2 (2.9%)	1 (4.0%)	0 (0.0%)	
Lung cancer subtype							
Adenocarcinoma	39 (51.3%)	9 (30.0%)	1 (33.3%)				^a^ *p* = 0.11658
Squamous cell carcinoma	22 (28.9%)	12 (40.0%)	0 (0.0%)				
Other lung neoplasms	15 (19.7%)	9 (30.0%)	2 (66.7%)				
Metastases							
No metastases	35 (46.1%)	14 (46.7%)	2 (66.7%)				^a^ *p* = 0.94658
To lymph nodes	30 (39.5%)	12 (40.0%)	1 (33.3%)				
Distant metastases	11 (14.5%)	4 (13.3%)	0 (0.0%)				

Notes: Light smokers are defined as people who smoke from >0 to <20 pack-years; moderate smokers are defined as people who smoke from ≥20 to <40 pack-years; and heavy smokers are defined as people who smoke ≥40 pack-years; NA—not available; statistical values of comparisons between individuals with CC, CT, and TT genotypes among cases are marked with an “a,” while among controls are marked with a “b”.

**Table 5:** Selected characteristics of lung cancer patients divided by their lung cancer subtype.

Variable	Adenocarcinoma [*n*, (%)] ^a,b^ *n* = 50 (44.6%)	Squamous Cell Carcinoma [*n*, (%)] ^a,c^ *n* = 35 (31.3%)	Other Lung Neoplasms [*n*, (%)] ^b,c^ *n* = 27 (24.1%)	*p*-Value (Pearson’s Chi-Square Test)
Age [years]				^a^ *p* = 0.74016
≤60	9 (18.0%)	6 (17.1%)	5 (18.5%)	^b^ *p* = 0.81861
>60	41 (82.0%)	29 (82.9%)	22 (81.5%)	^c^ *p* = 0.61571
Gender				^a^ *p* = 0.06694
Male	29 (58.0%)	27 (77.1%)	15 (56.6%)	^b^ *p* = 0.83614
Female	21 (42.0%)	8 (22.9%)	12 (44.4%)	^c^ *p* = 0.07140
Smoking status				
Never smoker	3 (6.0%)	0 (0.0%)	0 (0.0%)	
Light smoker	4 (8.0%)	3 (8.6%)	0 (0.0%)	^a^ *p* = 0.35977
Moderate smoker	17 (34.0%)	11 (31.4%)	5 (18.5%)	^b^ *p* = 0.33032
Heavy smoker	11 (22.0%)	12 (34.3%)	6 (22.2%)	^c^ *p* = 0.49688
NA	15 (30.0%)	9 (25.7%)	16 (59.3%)	
Metastases				
No metastases	26 (52.0%)	15 (42.9%)	12 (44.4%)	^a^ *p* = 0.57388
To the lymph nodes	20 (40.0%)	18 (51.4%)	6 (33.3%)	^b^ *p* = 0.01420
Distant	4 (8.0%)	2 (5.7%)	9 (22.2%)	^c^ *p* = 0.00701

Notes: Light smokers are defined as people who smoke from >0 to <20 pack-years; moderate smokers are defined as people who smoke from ≥20 to <40 pack-years; and heavy smokers are defined as people who smoke ≥40 pack-years; NA—not available. Comparisons between adenocarcinoma and squamous cell carcinoma patients are denoted by an “a”, while comparisons between adenocarcinoma and patients with other lung neoplasms are represented with a “b”, whereas comparisons between squamous cell carcinoma and patients with other lung neoplasms are denoted by a “c”.

**Table 6:** The effect of each variable on the risk of lung cancer when compared to healthy individuals.

Variable	Lung Cancer Patient vs. Control	
	OR (95% CI)	*p*-Value
Gender–Male	2.03 (1.17–3.52)	0.011458
Age	1.23 (1.16–1.30)	<0.000001
Pack-years	1.15 (1.10–1.19)	<0.000001
MMP-2 [ng/mL]	0.96 (0.95–0.97)	<0.000001
*MMP-2*-735 CC	5.39 (0.62–47.17)	0.238504
*MMP-2*-735 CT	7.22 (0.78–67.14)	0.072836
MMP-9 [ng/mL]	1.01 (1.00–1.01)	<0.000001
*MMP-9*-1562 CC	1.45 (0.31–6.70)	0.757914
*MMP-9*-1562 CT	1.60 (0.33–7.83)	0.548801

Abbreviation: OR—odds ratio; and CI—confidence interval.

**Table 7:** MMP-2 concentrations by the MMP-2-735C/T genotype, and MMP-9 concentrations by the MMP-9-1562C/T genotype observed in the non-smoking controls, smoking controls, and lung cancer patients divided by lung cancer subtype.

	MMP-2 [ng/mL]			MMP-9 [ng/mL]		
	*MMP-2*-735C/T Genotype			*MMP-9*-1562C/T Genotype		
	CC	CT	TT	CC	CT	TT
Non-smoking control (NSC)						
	(*n* = 39)	(*n* = 5)	(*n* = 3)	(*n* = 35)	(*n* = 10)	(*n* = 2)
Mean ± SEM	204.04 *^,a,b,c^ ± 5.43	237.00 *^,a’,b’,c’^ ± 10.01	207.54 ± 3.97	358.74 ± 23.19	452.62 ^A’,B’,C’^ ± 82.74	358.70 ± 32.02
Median	208.01	239.60	207.41	312.41 ^A,B,C^	396.93	358.70
Min–Max	145.21–275.71	207.97–269.46	200.74–214.47	192.94–697.64	152.14–941.59	326.68–390.72
Smoking control (SC)						
	(*n* = 37)	(*n* = 12)	(*n* = 2)	(*n* = 34)	(*n* = 14)	(*n* = 2)
Mean ± SEM	216.56 ^d,e,f^ ± 7.37	224.34 ^d’,e’,f’^ ± 14.69	190.92 ± 47.38	385.67 ^#,D,E,F^ ± 26.31	562.80 ^#,D’,E’,F’^ ± 45.55	648.57 ± 139.44
Median	215.81	217.85	190.92	378.48	519.45	648.57
Min–Max	134.65–317.92	148.72–318.04	143.54–238.30	148.51–633.65	317.65–840.55	509.13–788.02
Adenocarcinoma (ADC)						
	(*n* = 39)	(*n* = 10)	(*n* = 0)	(*n* = 39)	(*n* = 9)	(*n* = 1)
Mean ± SEM	157.69 **^,a,d^ ± 5.75	126.37 **^,a’,d’,g’^ ± 8.41		959.95 ^D^ ± 83.76	887.55 ^A’,D’^ ± 108.93	1307.07
Median	154.72	128.25		936.72A	779.53	1307.07
Min–Max	94.93–237.57	90.70–175.39		73.03–2143.81	654.54–1310.73	
Squamous cell carcinoma (SqCC)						
	(*n* = 28)	(*n* = 7)	(*n* = 0)	(*n* = 22)	(*n* = 12)	(*n* = 0)
Mean ± SEM	146.11 ^b,e^ ± 5.69	130.43 ^b’,e’,h’^ ± 12.81		880.26 ^E^ ± 79.06	819.39 ^B’,E’^ ± 124.75	
Median	142.00	137.85		778.66B	817.61	
Min–Max	90.70–209.82	81.92–180.09		403.44–1632.66	295.32–1514.18	
Other lung neoplasms (OLN)						
	(*n* = 16)	(*n* = 9)	(*n* = 1)	(*n* = 15)	(*n* = 9)	(*n* = 2)
Mean ± SEM	138.05 ^c,f^ ± 11.62	164.48 ^c’,f’,g’,h’^ ± 7.51	231.50	821.64 ^##,F^ ± 98.62	928.88 ^##,C’,F’^ ± 154.40	1527.66 ± 343.10
Median	134.93	164.78	231.50	932.81C	802.18	1527.66
Min–Max	59.16–227.51	133.53–200.94		104.94–1322.75	413.85–1801.21	1184.56–1870.75

Notes: SEM—standard error of the mean; statistical significances, Student’s *t*-test: * *p* = 0.041479 between the NSCs with the CC and CT genotypes (*MMP-2*-735C/T) in the MMP-2 concentration, ** *p* = 0.013222 between the ADC patients with the CC and CT genotypes (*MMP-2*-735C/T) in the MMP-2 concentration, ^a^ *p* < 0.000001 between NSCs and ADC patients with *MMP-2*-735CC genotype in MMP-2 concentration, ^a’^ *p* = 0.000002 between the NSCs and ADC patients with the *MMP-2*-735CT genotype in the MMP-2 concentration, ^b^ *p* < 0.000001 between the NSCs and SqCC patients with the *MMP-2*-735CC genotype in the MMP-2 concentration, ^b’^ *p* = 0.000115 between the NSCs and SqCC patients with the *MMP-2*-735CT genotype in the MMP-2 concentration, ^c^ *p* < 0.000001 between the NSCs and OLN patients with the *MMP-2*-735CC genotype in the MMP-2 concentration, ^c’^ *p* = 0.000087 between the NSCs and OLN patients with the *MMP-2*-735CT genotype in the MMP-2 concentration, ^d^ *p* < 0.000001 between the SCs and ADC patients with the *MMP-2*-735CC genotype in the MMP-2 concentration, ^d’^ *p* = 0.000023 between the SCs and ADC patients with the *MMP-2*-735CT genotype in the MMP-2 concentration, ^e^ *p* < 0.000001 between the SCs and SqCC patients with the *MMP-2*-735CC genotype in the MMP-2 concentration, ^e’^ *p* = 0.000456 between the SCs and SqCC patients with *MMP-2*-735CT genotype in the MMP-2 concentration, ^f^ *p* = 0.000001 between the SCs and OLN patients with the *MMP-2*-735CC genotype in the MMP-2 concentration, ^f’^ *p* = 0.036566 between the SCs and OLN patients with the *MMP-2*-735CT genotype in the MMP-2 concentration, ^g’^ *p* = 0.003790 between the ADC and OLN patients with the *MMP-2*-735CT genotype in the MMP-2 concentration, ^h’^ *p* = 0.029928 between the SqCC and OLN patients with the *MMP-2*-735CT genotype in the MMP-2 concentration, ^#^ *p* = 0.000936 between the SCs with the CC and CT genotypes (*MMP-9*-1562C/T) in the MMP-9 concentration, ^##^ *p* = 0.023315 between the OLN patients with the CC and CT genotypes (*MMP-9*-1562C/T) in the MMP-9 concentration, ^A’^ *p* = 0.006470 between the NSCs and ADC patients with the *MMP-9*-1562CT genotype in the MMP-9 concentration, ^B’^ *p* = 0.026914 between the NSCs and SqCC patients with the *MMP-9*-1562CT genotype in the MMP-9 concentration, ^C’^ *p* = 0.012350 between the NSCs and OLN patients with the *MMP-9*-1562CT genotype in the MMP-9 concentration, ^D^ *p* < 0.000001 between the SCs and ADC patients with the *MMP-9*-1562CC genotype in the MMP-9 concentration, ^D’^ *p* = 0.004315 between the SCs and ADC patients with the *MMP-9*-1562CT genotype in the MMP-9 concentration, ^E^ *p* = 0.000020 between the SCs and SqCC patients with the *MMP-9*-1562CC genotype in the MMP-9 concentration, ^E’^ *p* = 0.004493 between the SCs and SqCC patients with the *MMP-9*-1562CT genotype in the MMP-9 concentration, ^F^ *p* = 0.000401 between the SCs and OLN patients with the *MMP-9*-1562CC genotype in the MMP-9 concentration, ^F’^ *p* = 0.015655 between the SCs and OLN patients with the *MMP-9*-1562CT genotype in the MMP-9 concentration, Mann–Whitney U test: ^A^ *p* < 0.000001 between the NSCs and ADC patients with the *MMP-9*-1562CC genotype in the MMP-9 concentration, ^B^ *p* < 0.000001 between the NSCs and SqCC patients with the *MMP-9*-1562CC genotype in the MMP-9 concentration, and ^C^ *p* = 0.000043 between the NSCs and OLN patients with the *MMP-9*-1562CC genotype in the MMP-9 concentration.

**Table 8:** Characteristics of the used ELISA kits.

ELISA Kit	Standard Curve	Intra-Assay Precision	Inter-Assay Precision	Minimum Detectable Dose (MDD)
Cotinine	5–100 ng/mL	4.6–8.6%		1 ng/mL
MMP-2	0.5–32 ng/mL	3.6–7.0%	6.5–7.0%	0.033 ng/mL
MMP-9	0.313–20 ng/mL	1.9–2.9%	6.9–7.9%	<0.156 ng/mL

**Table 9:** Protocol of the designed and utilized PCR-RFLP method for *MMP-2*-735C/T (rs2285053) and *MMP-9*-1562C/T (rs3918242) polymorphism analyses.

	*MMP-2*-735 C/T	*MMP-9*-1562 C/T
Amplification of the promoter regions using PCR		
PCR Mix: (given amounts are calculated for one reaction)	Forward primer: 0.6 μL Reverse primer: 0.6 μL Gold Taq polymerase (5 U/μL): 0.2 μL 10× Gold buffer: 2 μL 25 mM MgCl_2_: 1.6 μL 10 mM dNTP Mix: 0.4 μL DNA: 2 μL PCR water: 12.6 μL	Forward primer: 0.6 μL Reverse primer: 0.6 μL Gold Taq polymerase (5 U/μL): 0.2 μL 10× Gold buffer: 2 μL 25 mM MgCl_2_: 1.6 μL 10 mM dNTP Mix: 0.4 μL DNA: 2 μL PCR water: 12.6 μL
PCR conditions:	Activation: 15 min at 95 °C 35 cycles of: Denaturation: 30 s at 95°C; Annealing: 30 s at 60 °C; Elongation: 30 s at 72 °C. Final elongation: 15 min at 72 °C Hold: ∞ at 4 °C	Activation: 15 min at 95 °C 35 cycles of: Denaturation: 30 s at 95 °C; Annealing: 30 s at 60 °C; Elongation: 30 s at 72 °C. Final elongation: 15 min at 72 °C Hold: ∞ at 4 °C
2. Digestion of the amplified DNA sequences with restriction enzymes		
Reaction Mix:	PCR product: 10 μL Anza™ 10(×) Buffer: 2 µL HinfI enzyme: 1 µL PCR water: 7 µL	PCR product: 10 μL 10(×) Buffer B: 2 µL PaeI enzyme: 1 µL PCR water: 18 µL
Reaction conditions:	Incubation: 16 h at 37 °C Inactivation: 20 min at 65 °C	Incubation: 16 h at 37 °C Inactivation: 20 min at 65 °C
3. Electrophoresis of the digested DNA fragments		
Agarose gel:	Agarose: 1.5 g	Agarose: 1.5 g
	TBE buffer 1(×): 100 mL	TBE buffer 1(×): 100 mL
	Gold DNA gel stain: 5 µL	Gold DNA gel stain: 5 µL
Electrophoresis conditions:	50 V for 5 min	50 V for 5 min
	120 V for 120 min	120 V for 120 min
Final products:	CC: 300 bp TT: 254 bp, 46 bp CT: 300 bp, 254 bp, 46 bp	CC: 435 bp TT: 247 bp, 188 bp CT: 435 bp, 247 bp, 188 bp

Notes: PCR-RFLP—polymerase chain reaction—restriction fragment length polymorphism; Mix—mixture; dNTP—deoxynucleoside triphosphate; 10(X)—ten times concentrated; V—voltage; CC—homozygous genotype CC; TT—homozygous genotype TT; CT—heterozygous genotype CT; and bp—base pair.

**Table 10:** Sequences of the used primers to detect the *MMP-2*-735C/T (rs2285053) and *MMP-9*-1562C/T (rs3918242) polymorphisms.

Genotype	Primer	Sequence
*MMP-2*-735 C/T	F primer:	5′-ATA GGG TAA ACC TCC CCA CAT T-3′
	R primer:	5′-GGT AAA ATG AGG CTG AGA CCT G-3′
*MMP-9*-1562 C/T	F primer:	5′-GCC TGG CAC ATA GTA GGC CC-3′
	R primer:	5′-TTC CTA GCC AGC CGG CAT C-3′

Notes: F primer—Forward primer; and R primer—Reverse primer.

## Data Availability

The data presented in this study are available on request from the corresponding author. The data are not publicly available due to privacy and ethical restriction.

## Supplementary Materials

The following supporting information can be downloaded at: https://www.mdpi.com/article/10.3390/ijms241310576/s1.
