# Supplementary material for: Analysis of MMP-2-735C/T (rs2285053) and MMP-9-1562C/T (rs3918242) Polymorphisms in the Risk Assessment of Developing Lung Cancer
Source: Int J Mol Sci. 2023 Jun 24;24(13):10576. doi: 10.3390/ijms241310576 (PMC10341518; doi:10.3390/ijms241310576)
Supplement: Supplementary file 1 [file ijms-24-10576-s001.zip › ijms-2415954-supplementary.pdf]

Supplementary materials

Table S1. Observed and expected MMP-2 -735C/T and MMP-9 -1562C/T genotype frequencies in controls and cases in accordance with Hardy-Weinberg equilibrium.

	Observed quantity			Expected quantity			*p* value (Pearson's chi-square test)
	CC	CT	TT	CC	CT	TT	
MMP-2 -735C/T (rs2285053)							
Control – overall	77	18	5	74	24	2	0.041271
Control – non-smokers	39	5	3	35	11	1	0.012686
Control – smokers	37	12	2	38	12	1	0.600091
Cases – overall	83	26	1	81	26	2	0.718193
Cases – adenocarcinoma	39	10	0	36	12	1	0.485565
Cases – squamous cell carcinoma	28	7	0	26	8	1	0.576778
Cases – other lung neoplasms	16	9	1	19	6	1	0.330751
MMP-9 -1562C/T (rs3918242)							
Control – overall	70	25	4	69	28	3	0.664254
Control – non-smokers	35	10	2	33	13	1	0.533840
Control – smokers	34	14	2	35	14	1	0.867274
Cases – overall	76	30	3	76	30	3	0.997984
Cases – adenocarcinoma	39	9	1	34	14	1	0.303539
Cases – squamous cell carcinoma	22	12	0	24	9	1	0.417721
Cases – other lung neoplasms	15	9	2	18	7	1	0.200349
